# Effects of non-landslide sampling strategies on machine learning models in landslide susceptibility mapping

**DOI:** 10.1038/s41598-024-57964-5

**Published:** 2024-03-26

**Authors:** Tengfei Gu, Ping Duan, Mingguo Wang, Jia Li, Yanke Zhang

**Affiliations:** 1 Faculty of Geography, Yunnan Normal University, Kunming, 650500 China; 2 Badong National Observation and Research Station of Geohazards, China University of Geosciences (Wuhan), Wuhan, 430074 China; 3 Yunnan Institute of Geological Surveying and Mapping Co., Ltd., Kunming, 650051 China; 4 Wuhan Tianjihang Information Technology Co., Ltd., Wuhan, 430074 China

**Keywords:** Landslide susceptibility, Non-landslide sample, Machine learning models, PU bagging, CatBoost, Natural hazards, Environmental impact, Information technology

## Abstract

This study aims to explore the effects of different non-landslide sampling strategies on machine learning models in landslide susceptibility mapping. Non-landslide samples are inherently uncertain, and the selection of non-landslide samples may suffer from issues such as noisy or insufficient regional representations, which can affect the accuracy of the results. In this study, a positive-unlabeled (PU) bagging semi-supervised learning method was introduced for non-landslide sample selection. In addition, buffer control sampling (BCS) and K-means (KM) clustering were applied for comparative analysis. Based on landslide data from Qiaojia County, Yunnan Province, China, collected in 2014, three machine learning models, namely, random forest, support vector machine, and CatBoost, were used for landslide susceptibility mapping. The results show that the quality of samples selected using different non-landslide sampling strategies varies significantly. Overall, the quality of non-landslide samples selected using the PU bagging method is superior, and this method performs best when combined with CatBoost for predicting (AUC = 0.897) landslides in very high and high susceptibility zones (82.14%). Additionally, the KM results indicated overfitting, displaying high accuracy for validation but poor statistical outcomes for zoning. The BCS results were the worst.

## Introduction

Landslides are a common geological hazard in mountainous areas. Due to the uncertainty and complexity of landslides, they are characterized by their wide distribution, high frequency, and rapid onset hazards^1,2^. Landslide susceptibility mapping (LSM) is the first step in preventing and mitigating landslides^3,4^.

Many types of empirical, deterministic, statistical, and machine learning models have been proposed for LSM^5–12^. Among them, machine learning models can best describe the nonlinear relationships between influencing factors and landslides and provide good predictive performance^13^. Based on the use of prior knowledge (learning the characteristics of landslide and non-landslide samples in advance), machine learning models are divided into unsupervised learning models and supervised learning models. The latter fully utilizes prior knowledge and achieves more accurate prediction results^14^.

The quality of sample data directly affects the prediction accuracy of models in LSM based on supervised learning. The known sample data in susceptibility mapping are landslide samples, which are obtained through field surveys or remote sensing interpretation^15,16^. However, non-landslide samples are unknown. If the selected non-landslide samples are noisy or insufficient regional representations, it will lead to insufficient learning ability of the model, thereby affecting the final prediction results. In addition, high-quality non-landslide samples can help the model learn the features of both classes in a more balanced way, improve the stability of the model, and reduce the fluctuations caused by different sampling results.

The problem of non-landslide sample selection has attracted the attention of some scholars and is mainly divided into qualitative and quantitative methods. A qualitative method involves random selection from areas where no landslides have occurred, such as selection from outside a 500 m buffer zone of landslides^17^, selection in areas where no landslides have occurred^18^, selection of river channels and areas with slopes less than 5°^19^, and selection in areas where landslides and river channels once occurred^20^, which are random in nature. Their receiver operating characteristic area under the curve ranges from 0.78 to 0.938, indicating significantly different prediction results and low stability. If the selected non-landslide samples are insufficient regional representations, it will lead to a model with insufficient learning ability. The latter two algorithms generally rely on only a small number of factors, which may exaggerate the impact of a factor on landslides and affect the final accuracy. The quantitative selection methods include statistical, unsupervised and semi-supervised methods, such as the use of the information quantity or frequency ratio for selection in very low and low susceptibility areas^21,22^, the use of K-mean clustering to select samples farthest away from landslide samples^23^, and the use of a semi-supervised multiple-layer perceptron to select samples in very low-susceptibility areas^24^. Unsupervised classification-based methods cannot obtain classification labels, and the similarity of the obtained sample set features can be very high, which can easily lead to overfitting. Statistical methods and semi-supervised classification methods account for the diversity of sample selection and make full use of prior knowledge, but in both methods, the selection area of non-landslide samples is determined based on one calculation; thus, the complexity of landslides is not fully considered, and the accuracy of sample selection may be affected.

To overcome the difficulty of selecting high-quality non-landslide samples, a semi-supervised non-landslide sample selection method based on positive-unlabeled (PU) bagging is proposed. The PU bagging algorithm is a semi-supervised iterative classification algorithm. Model training is based on randomly sampling points from an unlabeled dataset multiple times. The final non-landslide sample selection is based on the comprehensive results of multiple model calculations, which provides high stability. Given the “no free lunch” theorem in machine learning, this study also focuses on the uncertainty issues brought by machine learning models^25^. Random forest (RF)^26^, support vector machine (SVM)^27^, and categorical boosting (CatBoost)^28^ models were selected for comparative analysis. Qiaojia County, Yunnan Province, China, is selected as the study area. First, the PU bagging algorithm is used to select non-landslide sample points and map landslide susceptibility. Then, buffer control sampling (BCS), as a qualitative method, and K-means (KM) clustering sampling with an unsupervised classification algorithms are selected for comparison to verify the effectiveness of the PU bagging algorithm. Finally, RF, SVM, and CatBoost models are used to map landslide susceptibility and verify the stability of the algorithm. Accurate and reliable landslide susceptibility mapping results are obtained.

## Study area and data

### Study area

**Figure 1:**
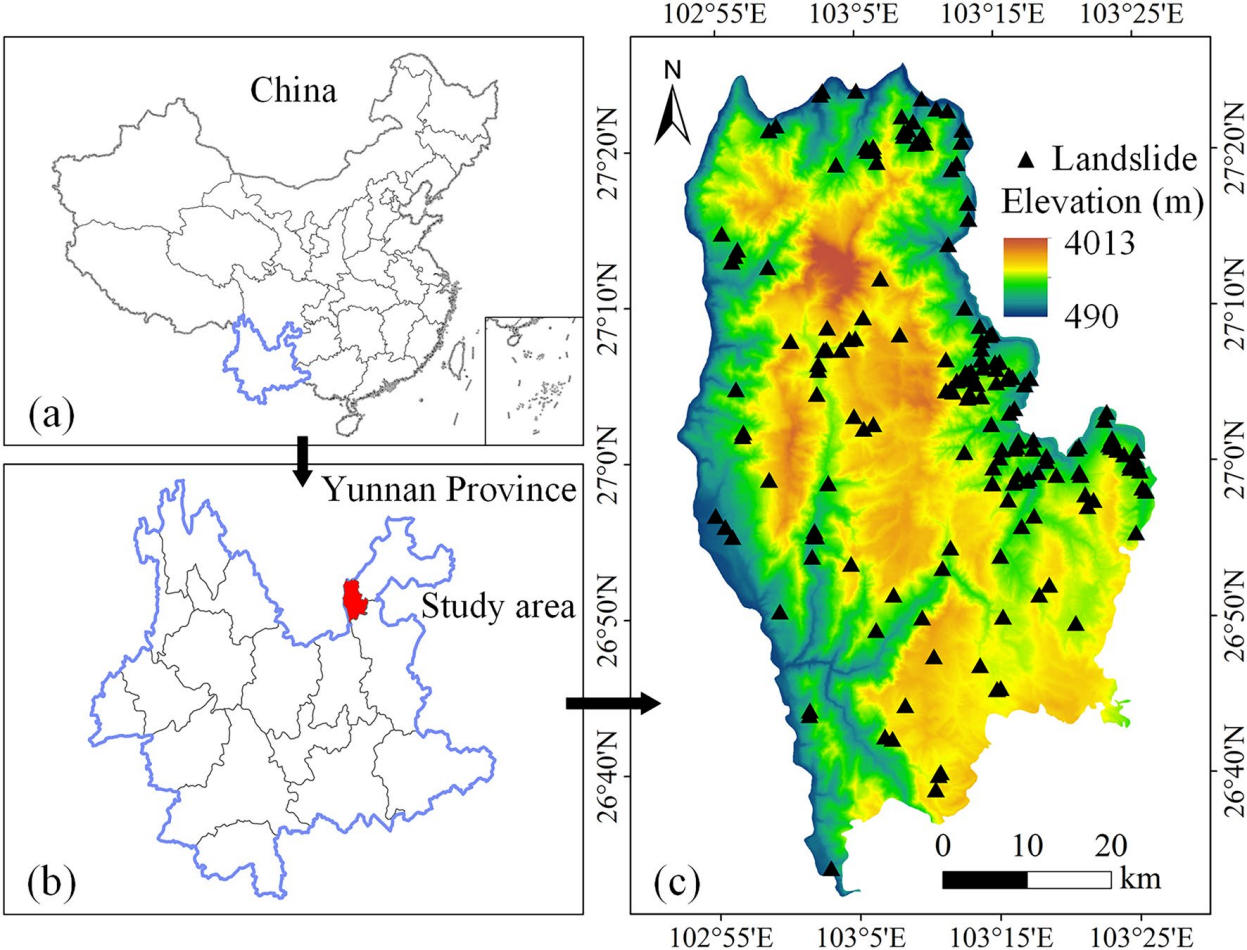
Location map of the study area (**a**) administrative boundaries map of China (**b**) administrative boundaries map of Yunnan Province, and (**c**) a digital elevation model of Qiaojia County where triangles show landslides of the study area. (Created using ArcGIS v10.2^29^).

Qiaojia County is located in the northeastern part of Yunnan Province, China, and belongs to the city of Zhaotong. Its geographical location is longitudes from 102°52′E to 103°26′E and latitudes from 26°32′N to 27°25′N, covering an area of 3245 km^2^ (Fig. 1). By the end of 2020, the county had 17 towns, 192 administrative villages, and a total population of approximately 625,000. Qiaojia County is bordered by rivers on three sides: the Jinsha River in the north and west and the Niulan River in the northeast. The terrain conditions, which have been affected by the erosion and dissolution of the Jinsha and Niulan rivers, are complex. With strong neotectonic movement, Qiaojia County is one of the key prevention areas for geological hazards in Yunnan Province.

### Data sources and impact factor processing

Selecting the appropriate impact factors is an important step in mapping the susceptibility of landslides^30,31^. In impact factor selection, we considered factors such as field investigations, study area characteristics, relevant literature, data availability, and acquired data quality. There were 15 impact factors selected from 5 aspects (topography and geomorphology, geological structure, hydrology and ecology, human activities, and earthquake conditions) for landslide susceptibility mapping: elevation, slope, aspect, profile curvature (PC), terrain ruggedness index (TRI), lithology, distance to faults (DTF), soil type, average annual precipitation (AAP), topographic wetness index (TWI), distance to rivers (DTRI), normalized difference vegetation index (NDVI), distance to roads (DTR), land use type, and peak ground acceleration (PGA).

**Figure 2:**
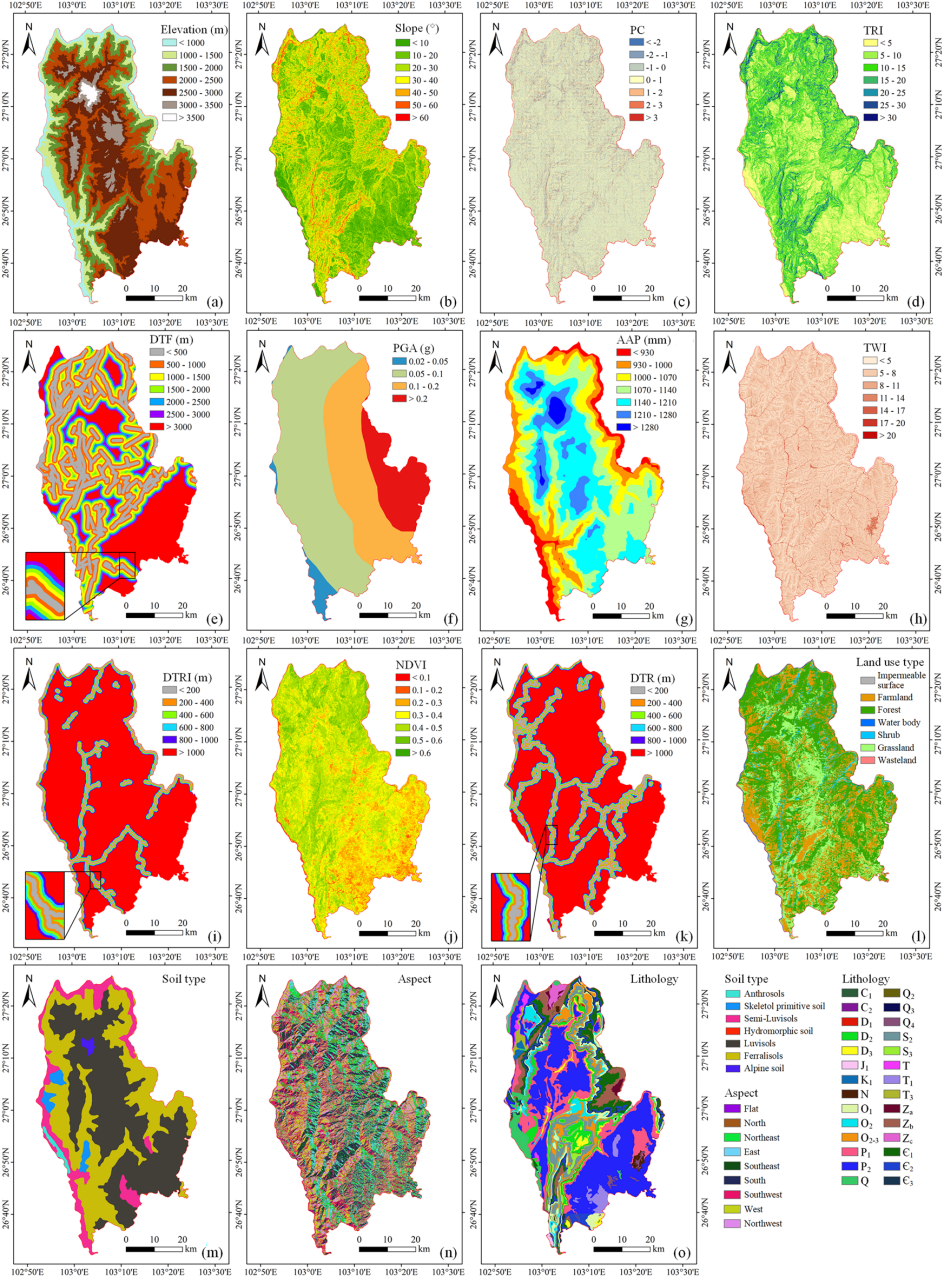
Thematic maps of landslide factors: (**a**) elevation, (**b**) slope, (**c**) profile curvature (PC), (**d**) terrain ruggedness index (TRI), (**e**) distance to faults (DTF), (**f**) peak ground acceleration (PGA), (**g**) average annual precipitation (AAP), (**h**) topographic wetness index (TWI), (**i**) distance to rivers (DTRI), (**j**) normalized difference vegetation index (NDVI), (**k**) distance to roads (DTR), (**l**) land use type, (m) soil type, (**n**) aspect, and (**o**) lithology. (Created using ArcGIS v10.2^29^).

The sources of impact factors were as follows. A digital elevation model (DEM) for the Qiaojia area was acquired from the China Geospatial Data Cloud site (http://www.gscloud.cn). Based on this DEM, the elevation, slope, aspect, PC, TRI and TWI were extracted. The lithology and faults were derived from the 1:200,000 geological map of China, and the lithology description is shown in Table 1. The NDVI was extracted from Landsat-8 OLI images (http://www.gscloud.cn). Soil type and precipitation were provided by the Resource and Environmental Science Data Center of the Chinese Academy of Sciences (http://www.resdc.cn). River and road data were obtained from Open Street Map (http://download.geofabrik.de/asia/china.html). Land use type data were extracted from 30 m land cover data (10.5281/zenodo.4417810)^32^. PGA was derived from the United States Geological Survey (https://earthquake.usgs.gov/earthquakes/eventpage/usb000rzmg/shakemap). Using ArcGIS software, all the influencing factors were converted into a raster data format with a reference scale of 30 m × 30 m and placed into the same projected coordinate system (Fig. 2).

**Table 1:** Description of stratum lithology in Qiaojia County.

Erathem	System	Series	Stratum symbol	Description
Cenozoic erathem	Quaternary system	Holocene series	Q_4_	Gravel, sand, sandy clay, clay, and humus
		Upper Pleistocene series	Q_3_	Moraine gravel
		Middle pleistocene series	Q_2_	Moraine conglomerate and limestone
		Unclassified series	Q	–
	Neogene system	Unclassified series	N	Conglomerate and carbonaceous claystone
Mesozoic erathem	Cretaceous system	Lower series	K_1_	Conglomerate
	Jurassic systerm	Lower series	J_1_	Clastic rock
	Triassic system	Upper series	T_3_	Mudstone, sandstone, and conglomerate
		Lower series	T_1_	Purple mudstone and siltstone shale
		Unclassified series	T	–
Upper paleozoic erathem	Permian System	Upper series	P_2_	Basalt
		Lower series	P_1_	Limestone, carbonate rock, sandstone, shale, bauxite, and carbonaceous shale sandwiched coal seam
	Carboniferous system	Middle series	C_2_	Carbonate rock
		Lower series	C_1_	Carbonate rock
	Devonian system	Upper series	D_3_	Carbonate rock
		Middle series	D_2_	Clastic rock, carbonate rock, and marl rock
		Lower series	D_1_	Clastic rock
Lower palaeozoic erathem	Silurian system	Upper series	S_3_	Shale
		Middle series	S_2_	Argillaceous rock, carbonate rock, and clastic rock
	Ordovician system	Middle and upper series	O_2-3_	Dolomite
		Middle series	O_2_	Clastic rock and carbonate rock
		Lower series	O_1_	Argillaceous rock, siltstone, and sandstone
	Cambrian system	Upper series	∈ _3_	Dolomite
		Middle series	∈ _2_	Shale, dolomite, clastic rock, argillaceous dolomite, and gypsum rock
		Lower series	∈ _1_	Fine sandstone, mudstone, dolomite, limestone, siltstone, argillaceous rock, and shale
Upper proterozoic erathem	Sinian system	Upper series	Z_b_	Dolomite
		Lower series	Z_a_	Quartz sandstone, gravel sandstone, feldspar rock, and clastic sandstone
Lower proterozoic erathem	Changchengian System	Huangcaoling group	Z_c_	Phyllite and slate

## Methodology

**Figure 3:**
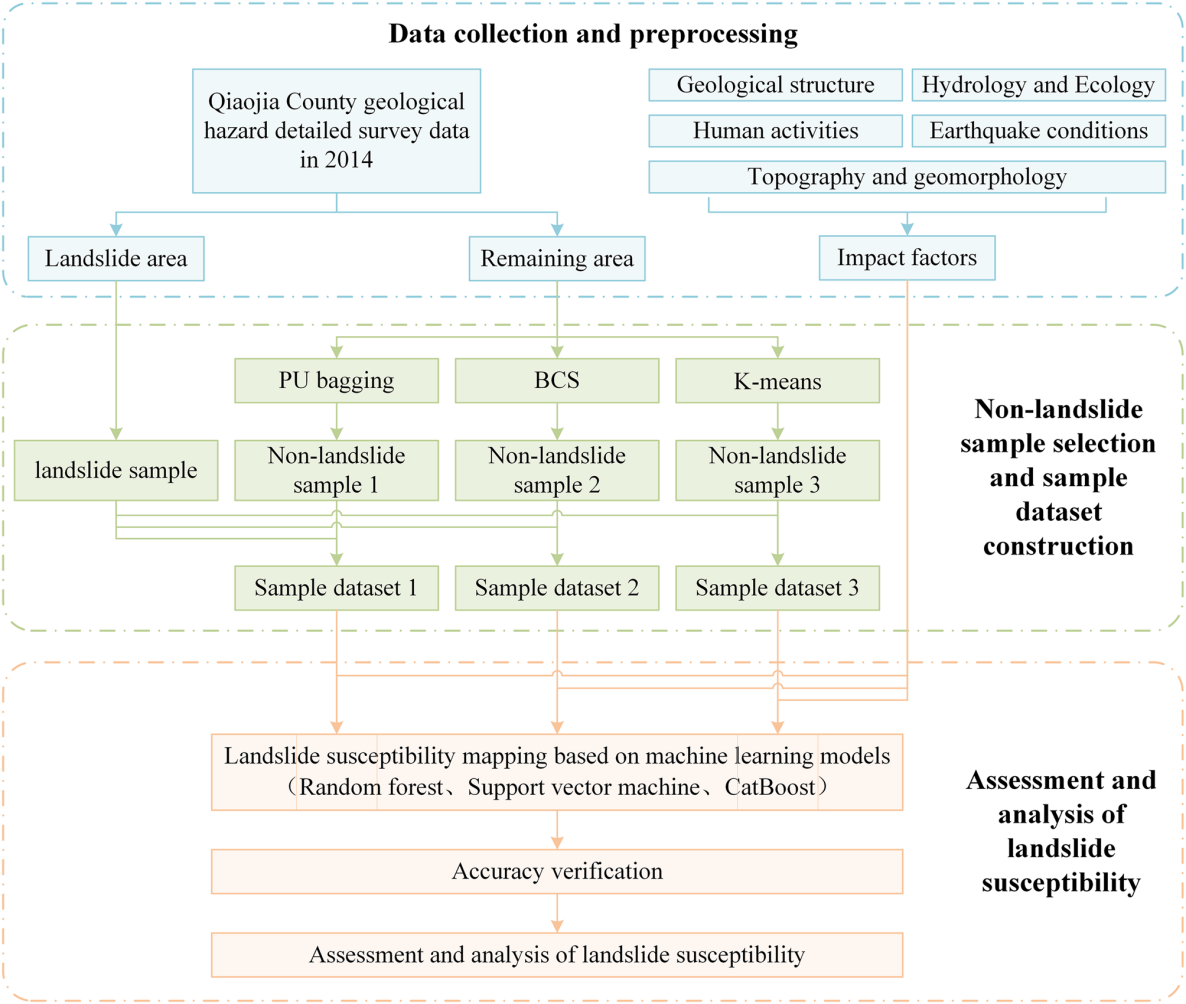
Flowchart of the methods.

Data from the 2014 landslide in Qiaojia County were taken as the research object. First, the study area was divided into landslide area and remaining area using the landslide data. Impact factors were collected and preprocessed from five aspects (topography and geomorphology, geological structure, hydrology and ecology, human activities, and earthquake conditions). Second, landslide samples are selected in the landslide area, and non-landslide samples are selected in the remaining area by PU Bagging, BCS and K-means, respectively, to build the sample data set. Finally, three sample data sets were combined with three machine learning models (RF, SVM, CatBoost) to map and evaluate landslide susceptibility, in which confusion matrix and ROC curve were used to verify accuracy. The flowchart of the research method is shown in Fig. 3.

### Non-landslide sampling methods

#### PU bagging

**Figure 4:**
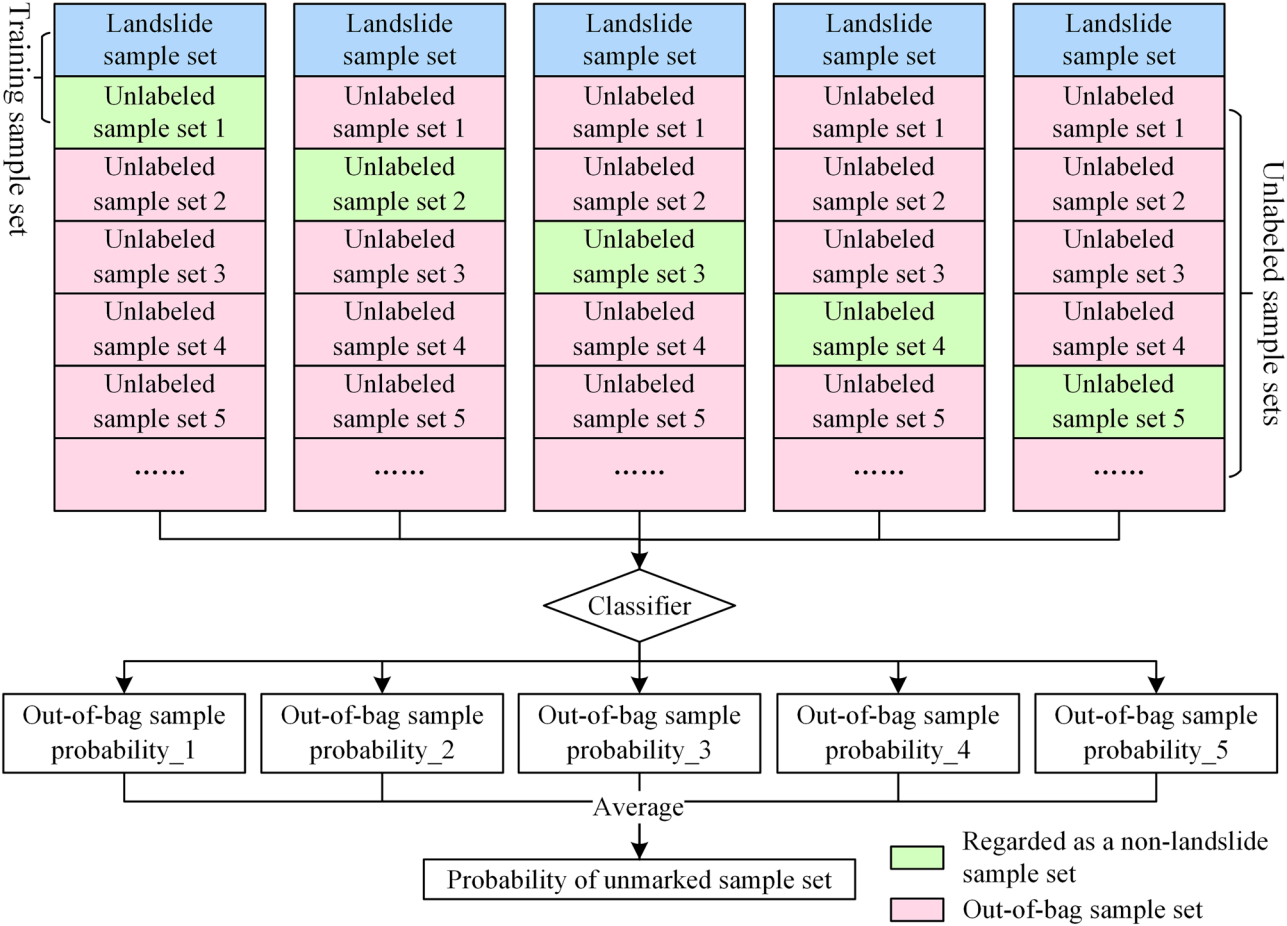
Flowchart of PU bagging.

PU bagging is a semi-supervised iterative classification algorithm^33,34^. The landslide sample data are learned, and then using the learned knowledge, the unlabeled samples are classified. The probability of landslides occurring in areas other than landslides is calculated through quantitative methods, and then non-landslide samples are selected in areas with low probability values, thereby improving the quality of the selected samples. The specific steps are as follows (Fig. 4):

1. Based on the landslide samples, an equal number of unlabeled samples are randomly selected from the unlabeled samples as non-landslide samples to construct a training sample set.
2. A decision tree is used to train the training sample set and generate a classifier.
3. A classifier is used to predict the samples that are not drawn from the unlabeled samples (out-of-bag samples) and treat the value as the probability that the sample belongs to the landslide samples.
4. Steps (1)–(3) are repeated to calculate the probability that all unlabeled samples belong to the landslide samples. The average probability obtained from the above multiple calculations was used as the final landslide probability for the unlabelled samples, aiming to mitigate prediction uncertainty and overfitting risks.

#### Buffer control sampling

**Figure 5:**
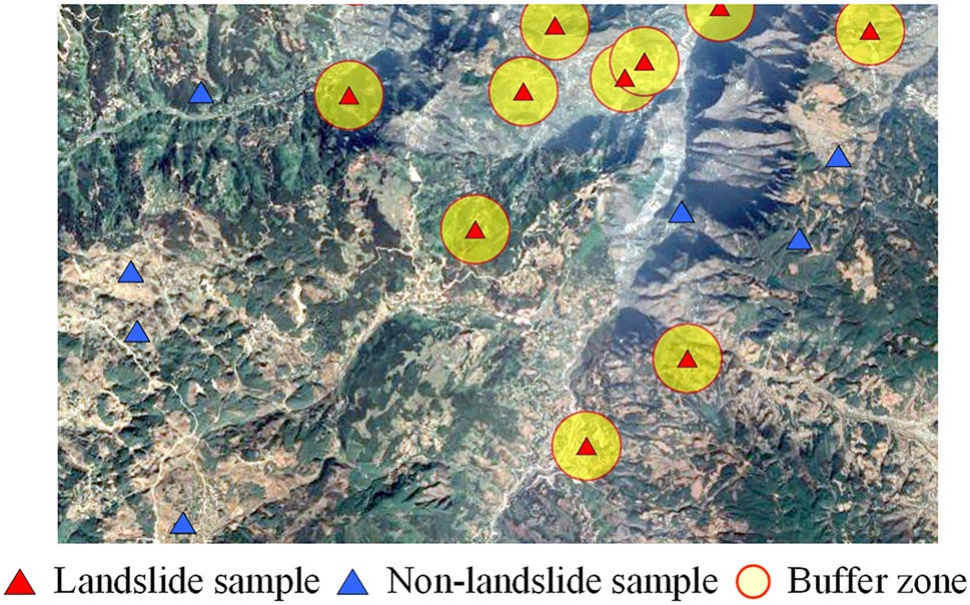
Schematic diagram of the BCS method.

The BCS method was inspired by the first law of geography^35^, which states that areas closer to landslides are more prone to landslides, and vice versa. The principle of this method in selecting non-landslide samples is simple, and it is easy to implement. In this method, which is the most commonly used method in landslide susceptibility mapping, the areas outside the landslide are considered non-landslide areas. Specifically, buffer zones are established around all landslide sample points, with the area outside the buffer zones considered non-landslide area. Samples from these areas, which are referred to as non-landslide samples, are randomly selected, as shown in Fig. 5. The size of the buffer distance is determined according to the scale of the landslide. However, areas outside the buffer zone may contain ancient or potential landslides. When a portion of potential landslide samples is misclassified as non-landslide samples, it increases the difficulty of learning for the model, leading to misguidance in the learning process and ultimately affecting the accuracy of the final predictions.

#### K-means clustering

The KM clustering method is an unsupervised classification algorithm that is applicable to the classification of unlabeled sample data^36^. The KM clustering method does not need to know the label (landslide or non-landslide) of each sample when training the model. It is based on classifying samples into different categories using the attribute characteristics of the impact factors. If there is a high degree of similarity within a category and a large difference between different categories, the classification result can be considered good.

The specific process of the KM clustering method in non-landslide sample selection is as follows: first, the study area is transformed into numerous individual samples, and the corresponding impact factor attribute eigenvalues of the samples are used as input data for the classification calculation. Then, the KM clustering method is used to classify the sample data into several classes. Finally, the number of landslide samples in each category is counted, and the category with the least number of landslide samples is selected as the data source for the non-landslide samples. The non-landslide samples selected using this method are highly similar to each other, resulting in them only representing a portion of the non-landslide areas and unable to fully reflect the complexity and variations of the non-landslide areas. When the representation of non-landslide samples is insufficient, the model may not adequately learn the characteristics of these samples, leading to overfitting by excessively learning from landslide samples during the training process.

### Landslide susceptibility mapping based on machine learning models

#### Random forest

RF is a very representative bagging ensemble algorithm consisting of multiple decision trees and is widely used in landslide susceptibility mapping^37^. It adopts a parallel method to establish multiple independent decision trees and then calculates the final prediction results based on the prediction results of each decision tree through voting principles. The construction of each tree relies on numerous a number of randomly selected impact factors, from which an optimal impact factor is selected when each node in the decision tree splits. The optimal impact factor can be determined by using the information entropy or Gini index, which indicates the correlation between the impact factor and the predicted result^38,39^. Compared with decision trees, RF has a stronger generalization ability and reduces the risk of overfitting by averaging decision trees.

#### Support vector machine

SVM is a machine learning model that follows the principle of structured risk minimization^40,41^. It converts the landslide sample data from a low-dimensional space to a high-dimensional space and converts the nonlinear classification problem in a low-dimensional space to a linear classification problem in a high-dimensional space. By finding an optimal hyperplane, the landslide and non-landslide data are spaced at a maximum distance apart. The kernel function is the core of the SVM and includes linear, polynomial, radial basis, and sigmoid functions. Linear or nonlinear classification problems can be satisfied with a variety of kernel functions.

#### CatBoost

The CatBoost model is a modification of the gradient boosting decision tree (GBDT) algorithm framework^42^. Compared with the mainstream GBDT (extreme gradient boosting and light gradient boosting machine) algorithms, the main advantage of the CatBoost model is that it deals with category-based factors using a target statistical approach without having to convert the category data into numerical data in advance. Second, CatBoost uses an ordered boosting framework to solve the gradient estimation bias problem and reduce the complexity of the algorithm. Finally, the complete binary tree used in the CatBoost model reduces the occurrence of overfitting and increases the speed of prediction^43^.

### Accuracy verification

A landslide problem is a binary classification problem (landslide or non-landslide), and the confusion matrix and ROC curve are the most commonly used evaluation indexes^44–47^. In the confusion matrix, the classification of the different sample categories can be clearly seen. We use several metrics to evaluate the performance of the model, including sensitivity, specificity, precision, accuracy, and F1-score. The five metrics vary between 0 and 1, and larger values indicate better model prediction performance^48^. The ROC curve is based on the confusion matrix and reflects the true positive rate (TPR) (sensitivity) and false positive rate (FPR) (1-specificity) under different thresholds. In the ROC curve approach, each inflection point has a corresponding FPR value as the x-coordinate and a TPR value as the y-coordinate^49^. The area under the curve (AUC) of the ROC curve is an indicator to measure the prediction effect of the model. The AUC value is between 0 and 1, and the larger the AUC value is, the better the prediction effect of the model^50,51^. The confusion matrix of landslides and non-landslides is shown in Table 2, and the equation for each metric is shown in Table 3.

**Table 2:** Landslide and non-landslide confusion matrix.

True situation	Prediction result	
	Landslide	Non-landslide
Landslide	TP	FN
Non-landslide	FP	TN

**Table 3:** Quantitative evaluation metrics for accuracy verification.

Metrics	Equation	Description
Sensitivity	$Sensitivity{ = }\frac{TP}{{TP + FN}}$	The ratio of the number of landslides successfully classified as landslides to the total number of landslides
Specificity	$Specificity{ = }\frac{TN}{{FP + TN}}$	The ratio of the number of successfully classified non-landslides to the total number of non-landslides
Precision	$Precision{ = }\frac{TP}{{TP + FP}}$	The ratio of correct landslide results to the number of landslide results predicted by the classifier
Accuracy	$Accuracy{ = }\frac{TP + TN}{{TP + FP + TN + FN}}$	The ratio of correctly predicted landslide and non-landslide samples to the total number of samples
F1-score	${\text{F}}1{\text{ - score}} = 2 \times \frac{Precision \times Sensitivity}{{Precision + Sensitivity}}$	Both precision and sensitivity metrics are considered together

## Results

### Sample dataset construction

First, the sample data needed for the model are prepared. The landslide samples were obtained from a detailed survey of geological hazards in 2014 by the Yunnan Institute of Geological Sciences, and a total of 188 landslide points were obtained. An equal number of non-landslide samples were selected to form a sample set, 70% of which were used as training samples, and the remaining 30% were used as test samples^52,53^. A total of 264 training samples and 112 test samples were obtained. The sampling results for the three non-landslide samples are as follows.

1. PU bagging method to construct non-landslide samples.

**Figure 6:**
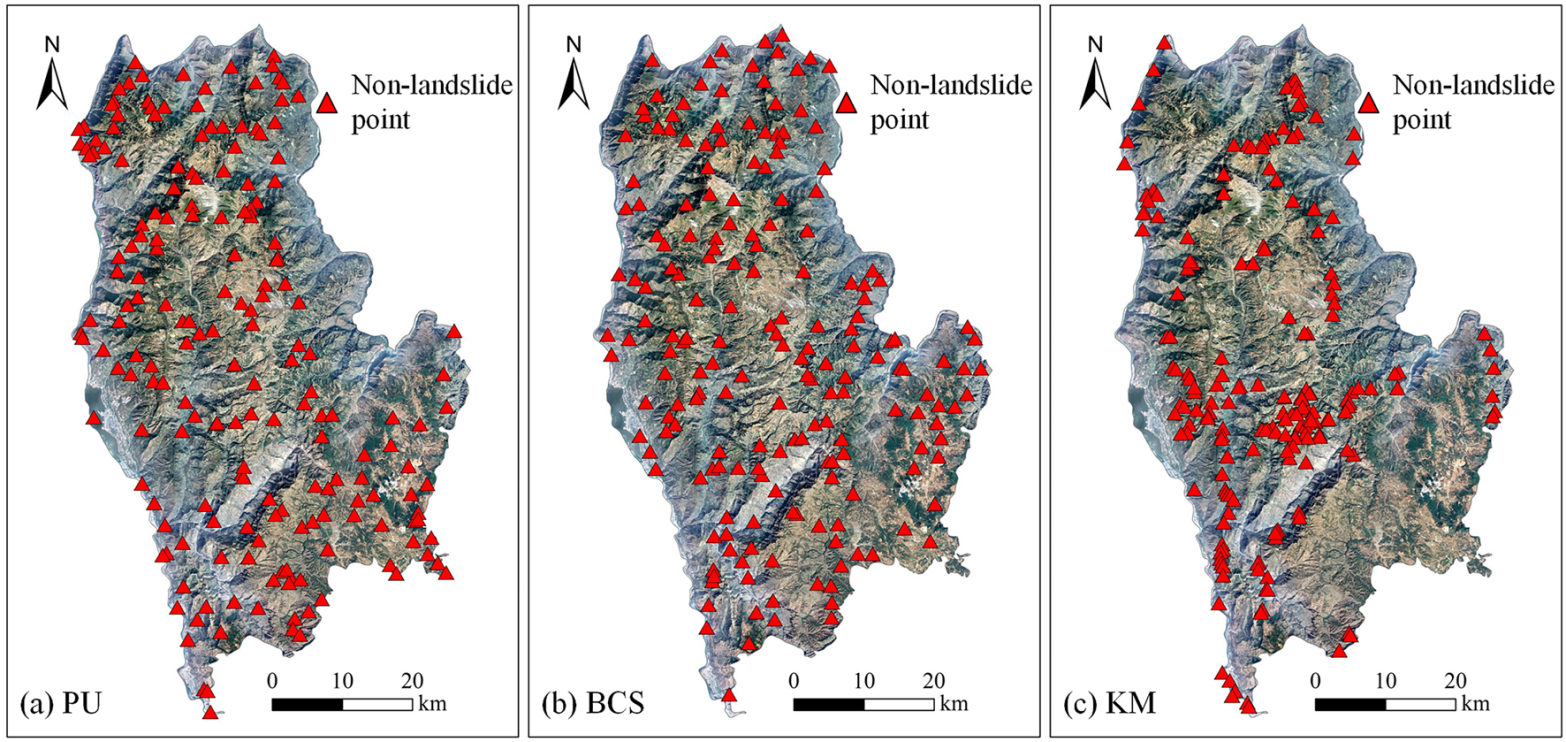
Sampling distribution map of three non-landslide samples: (**a**) result of PU bagging (PU), (**b**) result of buffer control sampling (BCS), and (**c**) result of K-means clustering (KM) (Created using ArcGIS v10.2^29^).

The grid corresponding to the study area was converted into single sample point data, and a total of 3,586,374 sample points were obtained. To improve the operational efficiency, 1 million sample points (188 landslide samples and 999,812 unlabeled samples) were extracted from all the data for the experiment. To ensure the accuracy of sample selection, the model was first trained. We selected 70% of the samples as training data and 30% of the samples as test data (56 samples were extracted from 188 landslide samples, and 56 samples were randomly selected from 999,812 unlabeled samples). Then, the trained model was used to calculate the probability value of the unlabeled samples being landslides. The above steps were repeated five times, and the average probability value of the five steps was used as the final probability value. Finally, non-landslide samples were selected by setting the probability threshold for landslide occurrence to 0.5, with samples exceeding this threshold classified as landslide samples and those equal to or below this threshold classified as non-landslide samples. The recall rate was used to verify the model training results, and it represents the ratio of the number of landslide samples that were correctly predicted to the total number of landslide samples. Because only landslide samples were known among all samples, this indicator was used as the evaluation basis. After calculations, the recall rate of the test samples was 0.95, indicating that the model provides high prediction ability for landslide samples. It can select non-landslide samples from the unlabeled sample set based on probability values. Finally, 272,008 landslide samples and 727,880 non-landslide samples were obtained from the unlabeled sample set. Additionally, 188 samples were randomly selected from the 727,880 samples regarded as non-landslide samples (Fig. 6a).

(2) Buffer control sampling method to construct non-landslide samples.

The BCS method was constructed on the basis of landslide samples. A total of 188 samples outside the 500 m buffer zone of the landslide points were randomly selected as non-landslide samples. To avoid the distances between the selected non-landslide samples from being too close to one another, the minimum distance threshold was set to 500 m (Fig. 6b).

(3) K-means clustering method to construct non-landslide samples.

The KM clustering algorithm and the PU bagging method use the same data, with 1 million samples for experiments. The attribute characteristics of all samples were substituted into the KM clustering algorithm, and the classification result was set to 5. To select a non-landslide sample set, the number of landslides in each category was counted. The number of landslides in each category and the relative landslide ratio results are shown in Table 4. The category with the least number of landslide samples and the lowest relative landslide ratio was selected as the source of non-landslide samples. It can be seen from the table that clustering result 3 met the requirements, the number of landslide samples was at least 9, and the relative landslide ratio was also the lowest. Therefore, 188 samples were randomly selected from clustering result 3 as non-landslide samples (Fig. 6c).

**Table 4:** Statistical table of k-means clustering analysis.

Clustering result	Number of sample points (pieces)	Landslide points (pieces)	Relative landslide ratio(10^–4^)
1	236,563	37	1.56
2	193,407	29	1.50
3	92,069	9	0.98
4	262,315	46	1.75
5	215,646	67	3.11

### Landslide susceptibility assessment

To enhance model performance, the best hyperparameters for each model were calculated using a Bayesian optimization algorithm^54^, and then the best hyperparameters were substituted into the models. All models were built using the Python language based on PyCharm software. Test samples were used to validate the prediction accuracy of the models.

#### Accuracy assessment

Five metrics, sensitivity, specificity, precision, accuracy, and F1-score, were used to validate the accuracy of the nine models (Table 5). For different non-landslide sample selection methods, the results showed significant differences. Comprehensive analysis of the five indicators shows that KM yields the highest values, followed by PU, and BCS produces the worst. For landslide prediction problems, using sensitivity (the proportion of successfully predicted landslides) to further measure the results yields the same results. However, during the statistical analysis of partitioning in section “Statistical analysis by zone”, it was noted that the KM prediction results display overfitting. Notably, the prediction accuracy of the PU method is superior, exhibiting a 0.089 higher accuracy than BCS. Regarding different machine learning models, for the PU bagging samples, the SVM model performs best in terms of the specificity and precision indicators. The CatBoost model yields the highest sensitivity, accuracy, and F1-score. Specificity reflects the effectiveness of the prediction results for non-landslide samples, and precision indicates the proportion of correctly predicted landslides in actual landslide forecasts. As landslides constitute highly hazardous disasters, while precision is important, greater attention should be given to the correct identification of landslides. The SVM identified 43 landslides, whereas CatBoost identified 47, indicating that the CatBoost model performs better. For the BSC samples, the performance of the SVM model was superior to that of both the RF and CatBoost models. The RF and CatBoost models exhibited strengths and weaknesses across different metrics. For the KM samples, the RF performed best in terms of specificity, precision, accuracy, and F1-score. CatBoost excels based on sensitivity, correctly predicting 94.6% of landslides.

**Table 5:** Model performance based on several evaluation metrics.

Non-landslide sampling method	Model	Sensitivity	Specificity	Precision	Accuracy	F1-score
PU bagging	RF	0.696	0.786	0.765	0.741	0.729
	SVM	0.768	**0.839**	**0.827**	0.804	0.796
	CatBoost	**0.839**	0.804	0.810	**0.821**	**0.825**
BCS	RF	**0.750**	0.643	0.677	0.696	0.712
	SVM	**0.750**	**0.696**	**0.712**	**0.723**	**0.730**
	CatBoost	0.696	0.679	0.684	0.688	0.690
KM	RF	0.929	**1.000**	**1.000**	**0.964**	**0.963**
	SVM	0.929	0.982	0.981	0.955	0.954
	CatBoost	**0.946**	0.946	0.946	0.946	0.946

Maximum values are in [bold].

**Figure 7:**
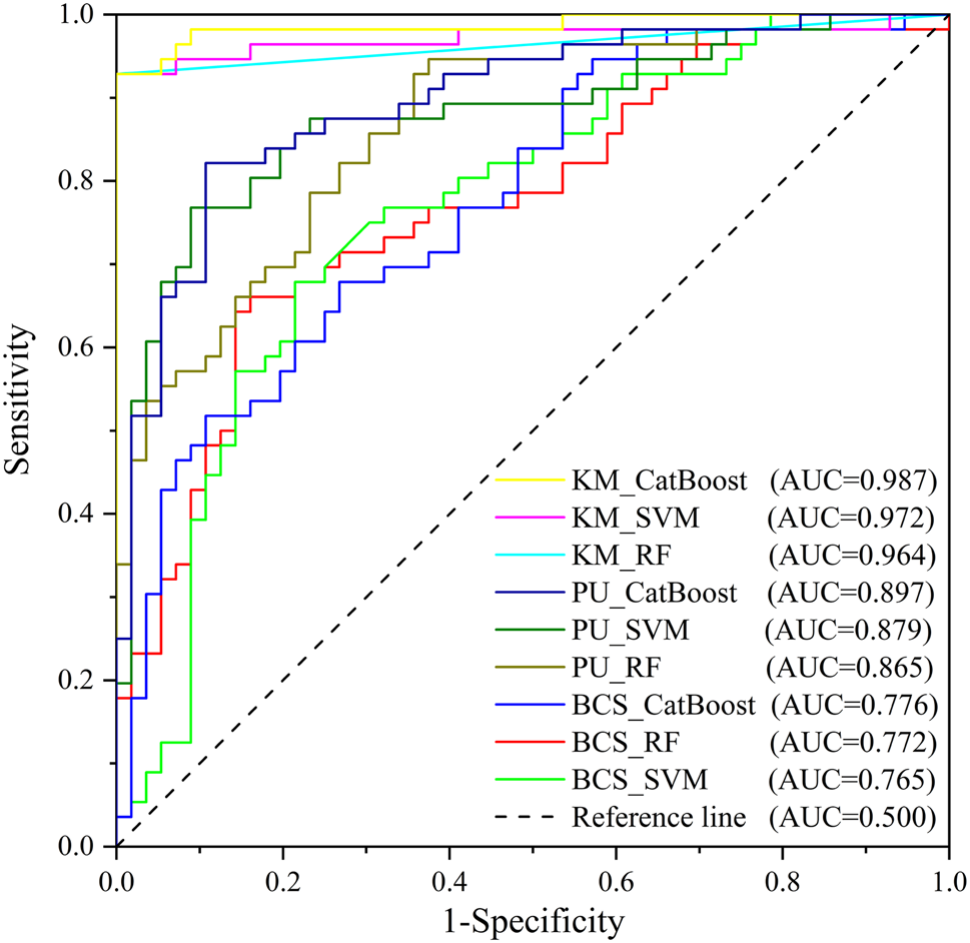
ROC curve results.

An accuracy assessment was performed using ROC curves, and the results of the ROC curves are shown in Fig. 7. Overall, the AUC values varied widely among models. From the perspective of non-landslide samples, the AUC values calculated using different non-landslide sampling strategies differed widely. The KM clustering results were the highest, the PU bagging results were the second highest, and the BCS results were the lowest. The values calculated using the same strategy differed less. This shows that different non-landslide sampling strategies have a large impact on the prediction results. From the perspective of machine learning models, CatBoost always displayed excellent prediction performance. In the sampling method using PU bagging, the AUC values of different models differed by a maximum of 0.032. Because the data used for accuracy verification were test samples, a partitioned statistical analysis was conducted to further explore the prediction performance of the three non-landslide sample selection methods in the study area.

#### Statistical analysis by zone

**Figure 8:**
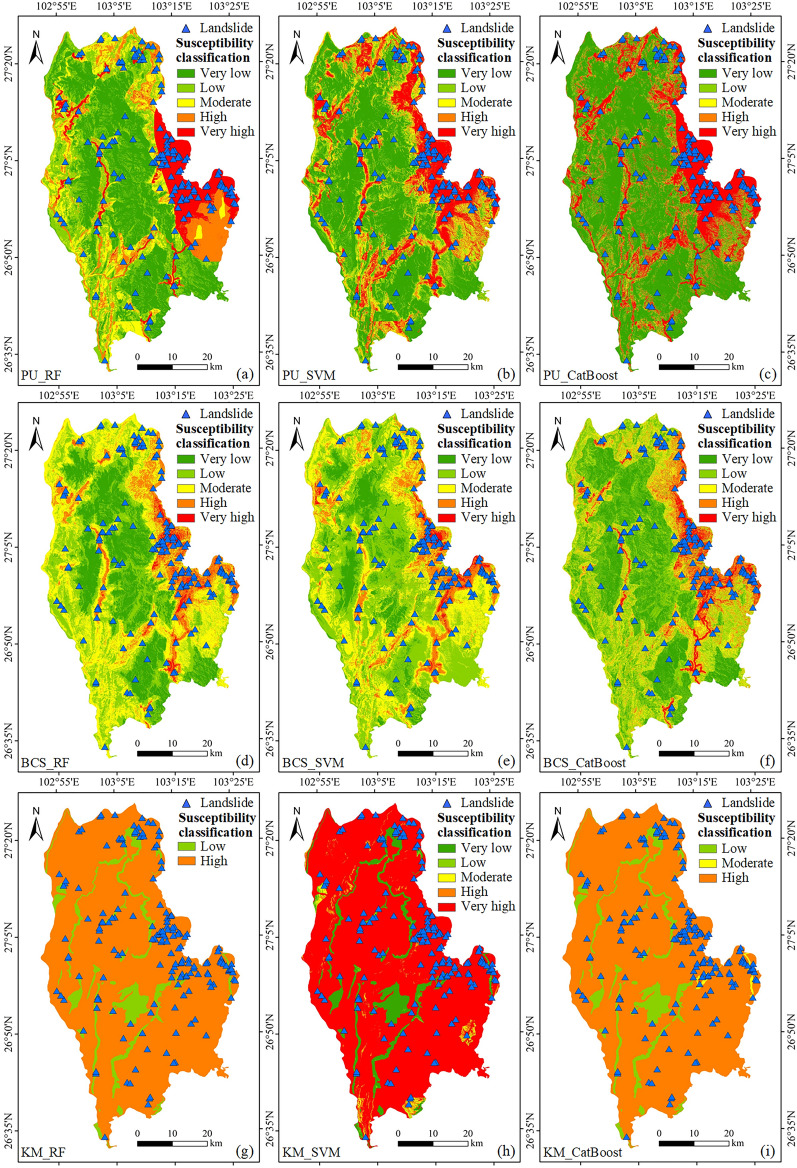
Landslide susceptibility classification map: (**a**) PU_RF, (**b**) PU_SVM, (**c**) PU_CatBoost, (**d**) BCS_RF, (**e**) BCS_SVM, (**f**) BCS_CatBoost, (**g**) KM_RF, (**h**) KM_SVM, and (**i**) KM_CatBoost. (Created using ArcGIS v10.2^29^).

The trained classifier was used to predict the study area and generate the landslide susceptibility prediction map in Qiaojia County. The landslide susceptibility probability map was divided into five classes according to the equal interval method^55^: very low (0–0.2), low (0.2–0.4), moderate (0.4–0.6), high (0.6–0.8), and very high (0.8–1) (Fig. 8).

**Figure 9:**
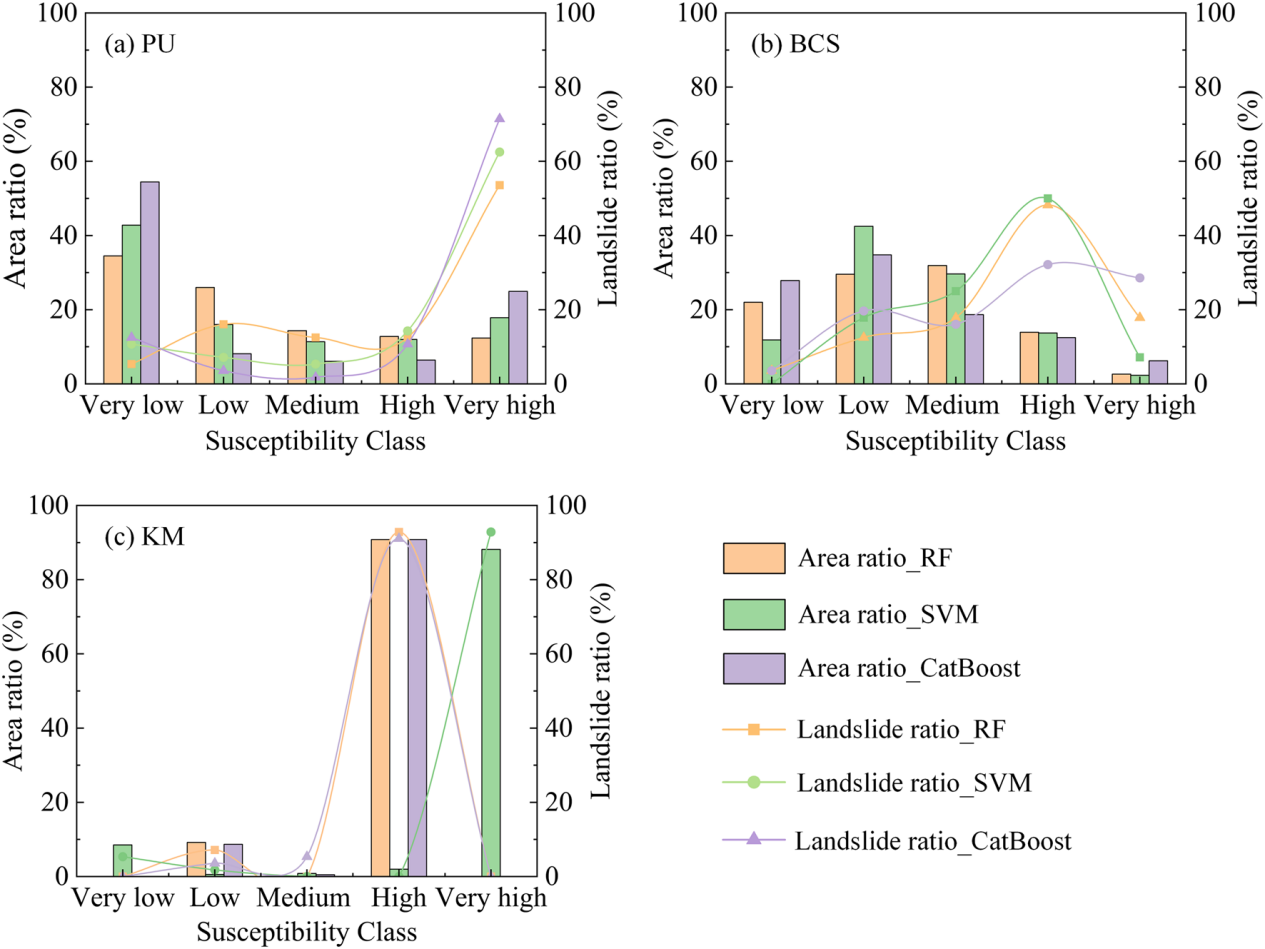
Susceptibility zonal statistics: (**a**) PU bagging, (**b**) buffer control sampling, (**c**) k-means clustering.

In the classification statistical analysis, two indicators, the area ratio and landslide ratio of each susceptibility zone, were used, and the statistical results are shown in Fig. 9. By observing the susceptibility partition map, we found that more than 90% of the area of the results obtained using the KM clustering method was classified as high or very high susceptibility areas. There were no very low or very high susceptibility areas in the KM_RF and KM_CatBoost maps. These prediction results are missing certain partitions, which obviously do not match the actual situation. The KM clustering method with the highest AUC value had the worst prediction results for the study area, with the illusion of better prediction accuracy, and the results of the remaining two non-landslide sampling methods were distributed among 5 classifications. From the landslide ratio, it was found that the BCS value suddenly decreased, and the PU value was the largest in the very high susceptibility area. In general, landslides should occur in the very high susceptibility zone. In both the very high and high susceptibility zones, the percentage of landslides based on the BCS method was less than 66.1%. In contrast, the percentage was higher than 66.1% based on the PU bagging method; the best value was 82.14%. Overall, the best landslide susceptibility results were obtained using the PU bagging method.

## Discussion

### The impact of different non-landslide sampling strategies on landslide susceptibility mapping

Training samples obtained by different non-landslide sampling strategies play an important role in the prediction of machine learning models. From the ROC accuracy verification and zonal statistics results in the paper, it was found that the non-landslide sampling strategy has an important impact on the prediction results. For landslide susceptibility mapping, only the landslide area sample data are known, and non-landslide samples are not directly available. The area beyond the landslide contains both non-landslide and potential landslide areas. Whether the selected non-landslide samples can represent the whole research area affects the model learning and generalization abilities.

**Figure 10:**
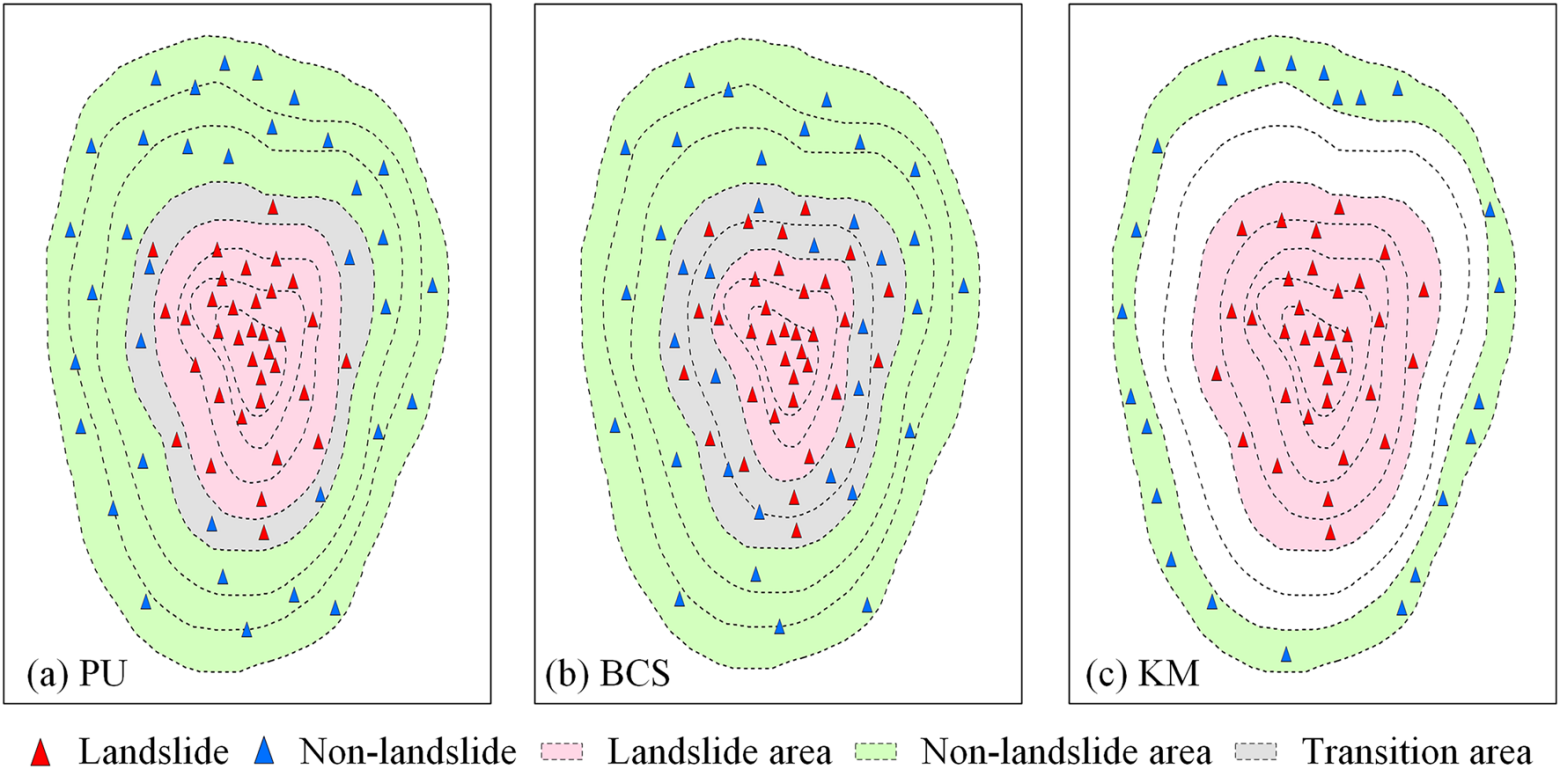
The spatial distribution of samples based on different non-landslide sampling methods: (**a**) PU bagging, (**b**) buffer control sampling, and (**c**) k-means clustering.

The spatial distribution of the sample dataset constructed based on PU bagging was inferred, as shown in Fig. 10a. The non-landslide samples were randomly selected from the sample points with a probability less than 0.5, the data quality of both landslide and non-landslide samples was relatively high, and the distribution was balanced. The quality of the training sample data was high, and the characteristics of the landslide and non-landslide samples were relatively clear for separation; therefore, the calculated AUC values were relatively high. When the selected training samples represent the research area, it facilitates the learning of the model. From the statistical results of landslide susceptibility classification obtained by the PU bagging method, it was found that its landslide susceptibility zoning also conforms to basic laws. Some of the landslides were in very low susceptibility areas because they occurred on slopes behind buildings. Models tend to overlook special cases when they learn general laws. Landslides are a kind of natural hazard, their occurrence law is not fixed, and there are certain special cases.

The spatial distribution of the sample dataset constructed based on BCS can be assumed, as shown in Fig. 10b. Non-landslide samples were randomly sampled outside the landslide buffer zone. They may contain many false non-landslide samples, and some of the non-landslide samples will have similar characteristics to the landslide samples. When some potential landslide samples are regarded as non-landslide samples, the learning difficulty of the model will increase, and it will become difficult to find regularity. When the number of fake non-landslide samples reaches a certain number, the learning process of the model will be misled. Thus, the model’s prediction ability will be insufficient, and the prediction accuracy will be reduced. By overlaying the landslide data with the landslide susceptibility map, the overall predictive ability of the BCS method was found to be insufficient from the susceptibility classes to which the landslide sites were assigned. Except for the high landslide occurrence area in the eastern part of Qiaojia County, landslides in other regions were not well predicted.

The spatial distribution of the sample dataset constructed based on KM clustering can be assumed, as shown in Fig. 10c. KM clustering uses distance as its similarity index. There is little similarity between different categories and high similarity within each category. The non-landslide samples selected according to this method have a high similarity, resulting in non-landslide samples that only represent a part of the non-landslide area. Thus, the complexity and changes in the non-landslide area cannot be fully reflected. The difference between the landslide and non-landslide samples was obvious, so the accuracy of the AUC value obtained under this training sample was very high, causing the illusion of high predictive power. The attribute features of the non-landslide samples were simple and easy for the model to learn, so the model preferred to learn the features of the landslide samples. In the process of model learning, there was little interference from the non-landslide samples, so the prediction results in the study area were easily overestimated. The results of susceptibility classification also proved that approximately 90% of the study area was predicted as high or very high susceptibility areas, but the high predictive ability was due to model overfitting. In addition, most of the areas in the partition results were only located in two of the susceptible partitions, which is obviously unreasonable.

### The impact of different machine learning models on landslide susceptibility mapping

We evaluate the performance of machine learning models using several metrics in a confusion matrix (sensitivity, specificity, precision, accuracy, and F1-score) and ROC curves. No single model is optimal for all metrics. In view of the overfitting exhibited by KM, the discussion focuses on the relationship between the different machine learning models and the two methods, PU bagging and BCS. For the RF model, PU bagging method is best under Specificity, Precision, Accuracy, F1-score metrics and BCS method is best in Sensitivity. For the SVM model, PU bagging performed best under all metrics. For the CatBoost model, also PU bagging performs best. Because the focus of different evaluation metrics in the confusion matrix is different, there may also be conflicts among the metrics. Therefore, the appropriate metrics should be selected according to the requirements in practical applications. From the analysis of the number of correctly predicted landslides (Sensitivity), CatBoost combined with PU bagging predicted the most (83.9%). In addition, the optimal results predicted among different machine learning models are not fixed, and this problem has been reported in several previous modeling studies^56^. This is because the classification criteria for models vary for different datasets and are influenced by the structure and underlying mechanisms of different models. The results show that the accuracy of SVM and CatBoost models is higher than RF. However, in the validation of ROC curves, the results display some regularity. The CatBoost model always maintains excellent prediction performance regardless of the sample dataset. CatBoost is a GBDT framework based on oblivious trees-based learners. This model can efficiently and effectively handle category-based factors and solve gradient bias and prediction shift problems. Thus, this approach reduces the occurrence of overfitting, and the accuracy and generalization ability of the model are improved. In addition, when evaluating model performance, the actual application of a model should be accounted for, and model performance should be analyzed comprehensively. In general, model performance is evaluated using training data or test data, but it is important to avoid generalized or biased results, such as in KM clustering.

## Conclusions

To overcome the difficulty of selecting high-quality non-landslide samples, an innovative hybrid model combining PU bagging and machine learning was proposed. In addition, BCS and KM were applied for comparative analysis. Based on landslide data from Qiaojia County, Yunnan Province, China, collected in 2014, three machine learning models, namely, RF, SVM, and CatBoost, were used for LSM. Then, the performance of different non-landslide sampling strategies was evaluated using the analysis results. The results of the study showed the following:

1. In machine learning models, there is a significant difference in the results obtained based on different non-landslide sampling strategies, indicating that the quality of selected non-landslide samples impacts the effectiveness of model training and prediction. However, the AUC values calculated from the same non-landslide sampling strategy displayed relatively minor differences.
2. The PU bagging method performed the best, with AUC values ranging from 0.865 to 0.897 across different machine learning models. Additionally, within very high and high susceptibility zones, this method successfully predicted 82.14% of landslides. However, the KM prediction results indicate overfitting, displaying high accuracy in validation but poor statistics-based zoning outcomes.
3. For different machine learning models, the CatBoost model displays excellent predictive performance. For the PU bagging samples, CatBoost identified the highest number of landslides (47). For the KM samples, CatBoost predicted the highest number of landslides (53). For the BSC samples, the performance of the SVM model was superior to that of both the RF and CatBoost models.

This study focuses on the selection of non-landslide samples, providing guidance for researchers when selecting samples. In cases in which definite positive samples (landslide samples) and uncertain negative samples (non-landslide samples) were analyzed using machine learning models, the PU bagging method proved to be adequate in producing reliable predictions.

## Data Availability

The datasets generated during and/or analysed during the current study are available from the corresponding author on reasonable request.
